# Injecting Drug Use History and Younger Age Worsen Adherence to Scheduled Hospital Visits in Glecaprevir and Pibrentasvir Therapy for Chronic Hepatitis C

**DOI:** 10.1155/ijh/5053151

**Published:** 2026-01-21

**Authors:** Seiichi Tawara, Asuka Watanabe, Junya Yamaguchi, Mai Omi, Tetsuro Miyazaki, Haruka Taguchi, Ryosuke Kiyota, Takuo Yamai, Shoichiro Kawai, Takuya Inoue, Takayuki Yakushijin

**Affiliations:** ^1^ Department of Gastroenterology and Hepatology, Osaka General Medical Center, Osaka, Japan

**Keywords:** glecaprevir, HCV, IDU, pibrentasvir

## Abstract

**Aim:**

The use of direct‐acting antivirals (DAAs) against the Hepatitis C virus (HCV) has rapidly expanded since their introduction. However, some patients with HCV infection may still not receive appropriate medical care. This study analyzed the characteristics and adherence of the population receiving therapy with two later‐generation DAAs, glecaprevir (GLE) and pibrentasvir (PIB), to investigate the clinical challenges associated with HCV treatment.

**Methods:**

A total of 141 consecutive patients who underwent GLE/PIB therapy for chronic HCV infection between December 2017 and June 2021 were enrolled. Patient backgrounds and adherence were retrospectively analyzed.

**Results:**

Median patient age was 61 years. Eighteen patients had a history of injecting drug use (IDU), accounting for 13% of the sample. At the end of treatment, three patients (2.1%) self‐discontinued hospital visits. The number of patients who self‐discontinued hospital visits gradually increased over time to 9 (6.4%) at 4 weeks after treatment, 16 (11.3%) at 12 weeks after treatment, and 24 (17.0%) at 24 weeks after treatment. The sustained viral response rate after 12 weeks, excluding patients who self‐discontinued hospital visits, was 96.8% (121/125). In a multivariate analysis, age < 60 years and a history of IDU were significant factors associated with the self‐discontinuation of hospital visits. The hazard ratio (HR) for those younger than 60 years old was 3.17 (*p* = 0.012), whereas the HR for those with a history of IDU was 2.41 (*p* = 0.036).

**Conclusions:**

History of IDU and younger age were significantly associated with poor adherence to GLE/PIB treatment.

## 1. Introduction

Recently, remarkable progress has been made in treating Hepatitis C virus (HCV) infections with direct‐acting antivirals (DAAs). Daclatasvir and asunaprevir, the first interferon‐free DAAs for HCV Genotype 1, were approved for use in Japan in July 2014 [1]. Subsequently, more DAAs were sequentially approved in Japan, such as sofosbuvir (SOF), ledipasvir (LDV), ombitasvir, paritaprevir, elbasvir, grazoprevir, beclabuvir, glecaprevir (GLE), pibrentasvir (PIB), and velpatasvir (VEL) [2–8]. Pioneering regimens were supplanted by the development of better regimens, and their sales were discontinued. GLE and PIB regimens were approved in Japan in September 2017. As of 2024, two pan‐genotypic regimens have persisted: GLE/PIB and SOF/VEL. In addition to their pan‐genotypic efficacy, these regimens are considered the first‐line treatment of choice for HCV due to their excellent safety profiles.

On World Hepatitis Day in 2017, the World Health Organization declared that by 2030, 90% of people with HCV infections will have been tested for the disease, and 80% of eligible patients will have accessed treatment [9]. This declaration may be realized due to the progress in treatment with DAAs. However, more than 1 million people in Japan are suspected of being infected with HCV [10]. Recently, it has become increasingly difficult to determine the exact number of HCV carriers in the country.

The most frequently prescribed DAA regimen in Japan was SOF/LDV, which was approved in August 2015. DAA prescriptions peaked sharply in 2015 [11]. Hence, the peak of DAA prescriptions occurred for early‐generation regimens, whereas prescriptions for later‐generation regimens gradually decreased. There should be a relationship between adherence and the time to treatment. If the number of patients with HCV infection is high, it should be recognized that they may not be receiving appropriate medical care. Possible reasons include not knowing about their infection or, even if their infection is known to them, poor adherence to medical care. This includes patients who are economically or socially vulnerable. To eradicate HCV, it is essential to provide treatment that emphasizes adherence. This study analyzed the characteristics and adherence of the population receiving GLE/PIB therapy, a later‐generation DAA regimen, and identified clinical challenges associated with HCV treatment in Japan.

## 2. Methods

### 2.1. Patients and Eligibility for GLE/PIB Therapy

One hundred forty‐one patients with chronic HCV infection were enrolled in this study. Patients underwent GLE/PIB (Mavyret; AbbVie GK, Tokyo, Japan) therapy between December 2017 and June 2021 at the Osaka General Medical Center.

Patients were asked to disclose any history of injecting drug use (IDU) and psychiatric disorders before GLE/PIB therapy was initiated, because we considered these characteristics as obstacles to the treatment completion of DAAs. If the patient had a history of IDU, we determined whether they had stopped and for how long. We also determined the patient′s arrest history related to IDU. In addition, we inspected their skin for marks indicative of IDU.

All patients underwent blood tests and abdominal ultrasounds to confirm the absence of liver cancer or decompensated cirrhosis. According to the Japan Society of Hepatology guidelines, GLE/PIB therapy should be administered for 8 weeks for chronic hepatitis and 12 weeks for compensated cirrhosis. This is the prescribed dosage according to the Japanese drug label. For GLE/PIB treatment, the diagnosis of chronic hepatitis or cirrhosis is based on the clinician′s judgment and does not require a pathological diagnosis from a liver biopsy. Therefore, clinicians in Japan may choose a 12‐week course of GLE/PIB for patients who show signs of cirrhosis, such as low serum albumin, low platelet counts, or prolonged prothrombin time.

### 2.2. Clinical and Biochemical Data

Clinical data included patient age, sex, and history of treatment for liver cancer or HCV infection. Blood and biochemical data included HCV‐RNA (SRL, Inc., Tokyo, Japan), HCV serogroup (SRL, Inc., Tokyo, Japan), platelet count, prothrombin time, serum albumin, total bilirubin, aspartate aminotransferase (AST), alanine aminotransferase, *γ*‐glutamyl transpeptidase (*γ*‐GTP), creatinine, estimated glomerular filtration rate (eGFR), alpha fetoprotein (AFP) level, and des‐*γ*‐carboxy prothrombin. Genotypes (SRL, Inc., Tokyo, Japan) were checked only when serogroups could not be identified.

### 2.3. Hospital Visit Protocol During and After GLE/PIB Therapy

**Figure 1:**
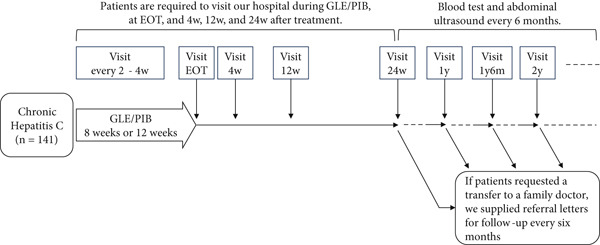
Protocol of hospital visits during and after GLE/PIB therapy. This figure illustrates the hospital visit protocol for GLE/PIB therapy for HCV infection. Patients continued to visit the hospital 6 months after GLE/PIB therapy. Subsequently, they underwent semiannual surveillance at the hospital or family doctor visits.

Patients were required to visit the Osaka General Medical Center during GLE/PIB treatment, at the end of treatment (EOT), and at 4, 12, and 24 weeks after treatment. They underwent blood tests and abdominal ultrasounds every 6 months thereafter. If patients requested a transfer to a family doctor, we supplied referral letters for follow‐up every 6 months (Figure 1).

Self‐discontinuation of hospital visits was defined as patients not attending their scheduled hospital visits and having no further hospital visits throughout the entire period from the start of treatment to the posttreatment follow‐up. The following cases did not constitute self‐discontinuation of hospital visits: (i) Although the patient did not visit on their scheduled day, they visited the hospital at a later date; (ii) the patient was referred to another hospital or clinic; (iii) the patient ceased to attend hospital visits, in agreement with their doctor, for a valid reason such as advanced age, worsened activities of daily living (ADLs), and treatment for other malignant diseases; and (iv) death. For patients who met these exclusion criteria, the hospital visit period was defined as the time up to their most recent visit before the event.

### 2.4. Statistical Analysis

Statistical analyses were conducted using JMP Pro software (Version 13.0; SAS Institute Inc., Cary, North Carolina, United States). Data are reported as medians with interquartile ranges. Univariable and multivariable Cox proportional hazards regression models were used to assess differences between categories. Crude hazard ratios were estimated using univariate Cox proportional hazards models, and adjusted hazard ratios were estimated using multivariate Cox proportional hazards models. Both ratios were reported with their corresponding 95% confidence intervals. Hospital visit data were analyzed using the Kaplan–Meier method, and groups were compared using the log‐rank test. Differences were considered statistically significant at *p* < 0.05.

## 3. Results

### 3.1. Patient Characteristics

Baseline patient characteristics are summarized in Table 1. The median age of the patients was 61 years. This study included 69 male and 72 female patients. Eighteen patients had a history of IDU (13%), and 23 patients lived with mental illness (17%).

**Table 1:** Demographic, clinical, serological, and virological characteristics of the 141 patients infected with HCV.

**Factor**	**Number or median [interquartile range]**
Age, year	61 [49–72]
Sex, man/woman	69/72
History of HCV treatment (DAAs or interferon), yes/no	19/122
History of liver cancer treatment, yes/no	7/134
History of injecting drug use, yes/no	18/123
Mental illness, yes/no	24/117
Serogroup (or Genotype) 1/2/3	60/80/1
HCV‐RNA, log IU/mL	6.4 [5.8–6.8]
Serum albumin, g/dL	4.1 [3.9–4.4]
Total bilirubin, mg/dL	0.7 [0.5–0.9]
Aspartate aminotransferase, U/L	32 [25–52]
Alanine aminotransferase, U/L	30 [20–54]
*γ*‐Glutamyl transpeptidase, U/L	28 [17–57]
Creatinine, mg/dL	0.79 [0.63–0.94]
eGFR, mL/min/1.73 m^2^	71.0 [56.3–83.4]
Prothrombin time, %	101.1 [90.6–108.4]
Platelet count, ´104/*μ*L	19.4 [14.9–23.7]
*α*‐fetoprotein, ng/mL	3.55 [2.26–5.54]
Des‐*γ*‐carboxy prothrombin, mAU/mL	22.8 [17.6–27.1]

Serogroups (or Genotypes) 1, 2, and 3 were identified in 60, 80, and 1 patient(s), respectively. Most patients had slightly elevated transaminase levels.

Patient characteristics of the 8‐week and 12‐week treatment groups are summarized in Table 2. There were significant differences between the two groups in sex, history of liver cancer treatment, HCV‐RNA levels, serum albumin levels, AST levels, *γ*‐GTP levels, creatinine, eGFR, prothrombin activity, platelet counts, and AFP levels. We observed that the patients in the 12‐week treatment group had more advanced liver fibrosis, poor renal function, and a history of liver cancer treatment.

**Table 2:** Demographic, clinical, serological, and virological characteristics of the 141 patients in the 8‐week and 12‐week treatment groups, respectively.

**Factor**	**Number or median [interquartile range]**		**p** **value**
	**8 weeks of treatment**	**12 weeks of treatment**	
Number of cases	113	28	
Age, year	58 [49–72]	67 [56–75]	0.1119
Sex, man/woman	48/65	21/7	0.0017
History of HCV treatment (DAAs or interferon), yes/no	12/101	7/21	0.0617
History of liver cancer treatment, yes/no	1/112	6/22	< 0.0001
History of injecting drug use, yes/no	16/97	2/26	0.2906
Mental illness, yes/no	22/91	2/26	0.0921
Serogroup (or Genotype) 1/2/3	47/66/0	13/14/1	0.1619
HCV‐RNA, log IU/mL	6.5 [5.9–7.1]	6.2 [4.8–6.6]	0.0345
Serum albumin, g/dL	4.2 [3.9–4.4]	3.8 [3.5–4.2]	< 0.0001
Total bilirubin, mg/dL	0.7 [0.5–0.9]	0.7 [0.5–1.2]	0.2716
Aspartate aminotransferase, U/L	31 [25–48]	37 [28–65]	0.0406
Alanine aminotransferase, U/L	28 [19–49]	33 [23–65]	0.0939
*γ*‐Glutamyl transpeptidase, U/L	25 [17–54]	42 [29–97]	0.0058
Creatinine, mg/dL	0.77 [0.63–0.89]	0.95 [0.74–3.71]	0.001
eGFR, mL/min/1.73 m^2^	73.0 [59.0–83.5]	57.4 [11.0–81.1]	0.005
Prothrombin time, %	102.6 [96.4–110.7]	87.4 [77.7–97.4]	< 0.0001
Platelet count, ´104/*μ*L	21.3 [17.6–25.5]	9.8 [8.6–11.5]	< 0.0001
*α*‐fetoprotein, ng/mL	3.23 [2.22–5.15]	5.10 [2.69–10.06]	0.0148
Des‐*γ*‐carboxy prothrombin, mAU/mL	22.9 [17.6–27.3]	22.1 [18.2–26.2]	0.9372

### 3.2. Hospital Visits and Sustained Viral Response Rate to 24 Weeks After GLE/PIB Therapy

Three patients (2.1%) did not visit the hospital for EOT. The number of patients lost to follow‐up gradually increased over time to 9 (6.4%) at 4 weeks after treatment, 16 (11.3%) at 12 weeks, and 24 (17.0%) at 24 weeks (Table 3). The overall sustained viral response rate after 12 weeks (SVR12), excluding patients who were lost to follow‐up, was 96.8% (121/125).

**Table 3:** HCV‐RNA detection and SVR rate in all patients by attendance of scheduled hospital visits.

**All cases**	**HCV-RNA detection**	**SVR rate excluding patients without a hospital visit**	**Rate of no hospital visit**
End of treatment, ND/detected/no hospital visit	136/2/3	98.6%	2.1%
After treatment			
Week 4, ND/detected/no hospital visit	130/2/9	98.5%	6.4%
Week 12, ND/detected/no hospital visit	121/4/16	96.8%	11.3%
Week 24, ND/detected/no hospital visit	113/4/24	96.6%	17.0%

The sustained viral response (SVR) rate of Serogroup (or Genotype) 1 after 4, 12, and 24 weeks, excluding patients who were lost to follow‐up, was 100% (Table 4). One patient with a positive signal at the EOT achieved SVR4, SVR12, and SVR24. Fragments of destroyed HCV‐RNA were detected at EOT. In contrast, the SVR rates for Serogroup (or Genotype) 2 after 4, 12, and 24 weeks, excluding patients who were lost to follow‐up, were 97.3% (73/75), 94.4% (68/72), and 94.1% (64/68), respectively (Table 5).

**Table 4:** HCV‐RNA detection and SVR rate in Serogroup (or Genotype) 1 patients by attendance of scheduled hospital visits.

**Serogroup (or Genotype) 1**	**HCV-RNA detection**	**SVR rate excluding patients without a hospital visit**	**Rate of no hospital visit**
End of treatment, ND/detected/no hospital visit	57/1^a^/2	98.3%	3.3%
After treatment			
Week 4, ND/detected/no hospital visit	56/0/4	100.0%	6.7%
Week 12, ND/detected/no hospital visit	53/0/7	100.0%	11.7%
Week 24, ND/detected/no hospital visit	48/0/12	100.0%	20.0%

^a^One patient with a signal positive at the end of treatment achieved SVR.

**Table 5:** HCV‐RNA detection and SVR rate in serogroup (or genotype) 2 patients by attendance of scheduled hospital visits.

**Serogroup (or Genotype) 2**	**HCV-RNA detection**	**SVR rate excluding patients without hospital visit**	**Rate of no hospital visit**
End of treatment, ND / Detected / no hospital visit	78 / 1 / 1	98.7%	1.3%
After treatment			
Week 4, ND / Detected / no hospital visit	73 / 2 / 5	97.3%	6.3%
Week 12, ND / Detected / no hospital visit	68 / 4 / 8	94.4%	10%
Week 24, ND / Detected / no hospital visit	64 / 4 / 12	94.1%	15%

### 3.3. Factors Associated With Self‐Discontinuation of Hospital Visit

Univariate analysis revealed thatage < 60 years, history of IDU, and history of mental illness were factors associated with the self‐discontinuation of hospital visits (Table 6). However, sex, serogroup, and history of HCV treatment showed no association. Age < 60 years and a history of IDU were significant factors in the multivariate analysis, in which there were three significant factors. The HR for patients younger than 60 years was 3.17 (*p* = 0.012), whereas the HR for an IDU history was 2.41 (*p* = 0.036). In addition, all patients with a history of liver cancer treatment or who received 12‐week GLE/PIB treatment continued their hospital visits. Therefore, these two factors were not associated with self‐discontinuation of hospital visits.

**Table 6:** Factors associated with self‐discontinuation of hospital visits.

**Factor**	**Category**	**Univariate analysis**		**Multivariate analysis**	
		**Hazard ratio (95% CI)**	**p** **value**	**Hazard ratio (95% CI)**	**p** **value**
Age, year	≤ 59, 60 ≤	4.31 (1.86–10.01)	0.0007	3.17 (1.29–7.76)	0.012
Gender	Man, woman	1.57 (0.77–3.19)	0.21		
Serogroup (or genotype)	1, 2	1.02 (0.50–2.09)	0.99		
History of HCV treatment	Yes, no	0.63 (0.19–2.07)	0.44		
History of injecting drug use	Yes, no	3.92 (1.79–8.57)	0.0006	2.41 (1.06–5.46)	0.036
Mental illness	Yes, no	2.89 (1.38–6.05)	0.005	1.69 (0.77–3.70)	0.19

There were significant differences in the Kaplan–Meier curves for age and IDU history. More than half of the patients aged < 60 years stopped visiting the hospital for follow‐up surveillance 2 years after treatment (*p* = 0.0002) (Figure 2a). All patients with a history of IDU stopped visiting the hospital after approximately 2 years after treatment (*p* = 0.0002) (Figure 2b).

Figure 2Rate of attendance for scheduled hospital visits by (a) age and (b) presence of IDU. There was a significant difference in attendance of scheduled hospital visits after GLE/PIB therapy according to (a) patient age and (b) the presence of IDU.

**Figure:**
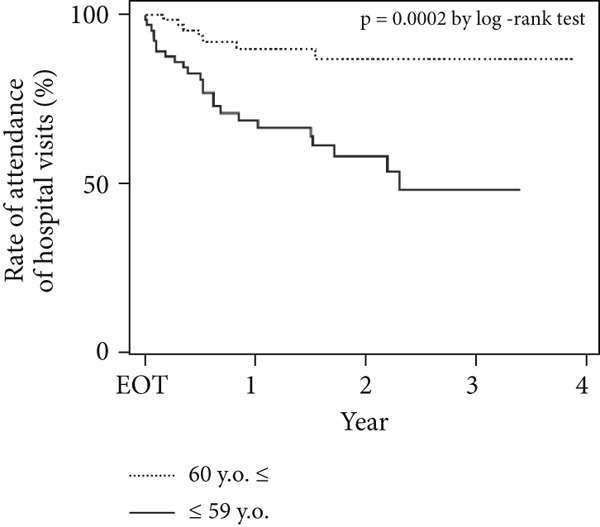
(a)

**Figure:**
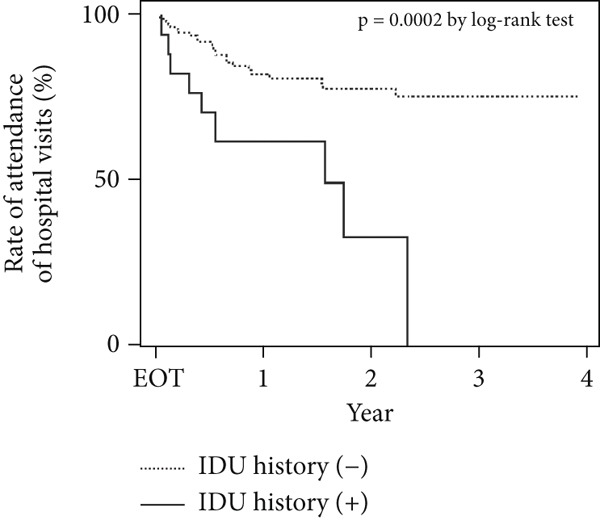
(b)

## 4. Discussion

Achieving SVR reduces the risk of hepatocellular carcinoma (HCC) in patients with HCV infection [12, 13]. This risk decreases over time [14]. However, even if SVR is achieved, cirrhosis is associated with a higher risk of HCC than noncirrhosis [15]. These findings suggest that all post‐SVR cases require follow‐up for several years, and patients with post‐SVR liver cirrhosis require long‐term follow‐up. Recently, DAAs have been shown to produce high SVR rates. A previous report showed that the SVR rates of GLE/PIB therapy were 97.7%–99.2% [16, 17]. The SVR rates in our cohort were comparable. Thus, HCV treatment must shift its focus from achieving a higher SVR rate to improving adherence to scheduled hospital visits for regular checkups after SVR.

Three patients (2.1%) in this study ceased hospital visits at the end of GLE/PIB treatment. Interestingly, all patients attended their scheduled hospital visit before the EOT and collected their prescriptions. This suggests that patients might have a strong desire to receive treatment, but they might not understand the importance of hospital visits after EOT. If patients stop visiting the hospital within 12 weeks after EOT, it is not possible to know their exact SVR rates. Improving adherence to follow‐up appointments after DAA treatment is therefore also essential for describing real‐world SVR rates more accurately.

Younger age was one of the factors associated with poor adherence to visits in this study. As mentioned above, the main purpose of check‐ups after SVR is to detect the incidence of HCC. Younger age makes it more difficult to predict the risk of developing cancer after treatment. However, older adults tend to have a greater fear of cancer based on their life experiences. It is likely that recognition of HCC risk after SVR differs depending on age. Another possible reason for this association is that young people may have professional commitments that make it difficult to attend check‐ups. However, no study has examined age‐related differences in compliance and adherence to follow‐up visits after achieving SVR. Future studies should examine the association between age and adherence.

Oze et al.′s cohort was from a different time period but in the same region as this study, and Genotype 1 accounted for 77% of patients [12]. In contrast, Genotype 2 accounted for 57% of patients in our study. Genotype 1, which is prevalent in postblood transfusion hepatitis, was once the dominant type of HCV infection in Japan. In recent years, the prevalence of Genotype 2 has increased in younger people and has become dominant, representing over 50% of HCV infections [18]. Blood transfusion is no longer a primary route for HCV infection. On the other hand, IDU is considered to be a major route for HCV infection. HCV clusters occur in communities of individuals who inject drugs [19]. Thus, a correlation may exist between IDU and Genotype 2 in Japan [18]. The high proportion of Genotype 2 in our cohort was consistent with the current clinical landscape in Japan.

IDU was an important factor in this study. Injecting drug users, often referred to as people who inject drugs (PWIDs), are socially vulnerable. HCV infection is often found in socially vulnerable groups such as PWIDs [20]. A substantial number of PWIDs live with HCV worldwide and are exposed to multiple adverse environmental conditions that increase health risks [21]. HCV treatment for PWIDs requires interventions against poverty, crime, stigma, and alienation from society [22]. In other words, if PWIDs do not receive social care, they will not adhere to their HCV treatment. In our study, we identified the challenges of HCV treatment in patients with a history of IDU.

Brown et al. demonstrated a relationship between medication adherence and alcohol use during the GLE/PIB treatment period [23]. Zamor et al. showed that adherence decreased with longer GLE/PIB treatment duration and reported that psychiatric disorders were associated with adherence below 80% during treatment [24]. Interestingly, addictive substances such as alcohol and injection drugs are factors influencing adherence in HCV treatment. In our study, we assessed adherence from treatment through posttreatment follow‐ups, covering a longer period than those previously reported. Darvishian reported that the loss‐to‐follow‐up rate in DAA treatment for Genotypes 1 and 3 was significantly higher among individuals aged under 60 years, those with a history of injection drug use, those on opioid substitution therapy, and patients with cirrhosis [25]. Clarifying the factors related to adherence in HCV treatment may contribute to improved treatment outcomes in the future.

A history of IDU and younger age suggest a risk of HCV reinfection. Patients with a history of IDU are more likely to return to living as PWIDs if their life expectancy is higher than expected. Prescribing DAAs each time an HCV reinfection occurs is economically wasteful. Education and adherence to checkups after SVR are essential for HCV treatment. Cooperation is needed among hospitals, drug rehabilitation facilities, local governments, public agencies, and police to educate and increase awareness and adherence among patients, including those who are PWIDs. If these organizations collaborate, patients should benefit from better HCV detection, treatment, and post‐SVR surveillance. Although the Ministry of Health, Labor and Welfare has recognized the public health concern posed by PWIDs with HCV, no organization has taken the initiative and assumed a leadership role to steer this cooperation. These problems must be overcome to eradicate HCV infection worldwide.

Our study has several limitations. First, it was a retrospective study. Further prospective validation studies are required to confirm these results. Second, because drug use is a crime in Japan, including both IDU and marijuana use, we need to consider the possibility that IDU numbers within our study were higher than reported by patients, as some patients were unlikely to confess their IDU history.

## 5. Conclusions

A history of IDU and younger age were significantly associated with poor adherence to GLE/PIB treatment.

**Nomenclature**
HCV: Hepatitis C virus
DAAs: direct‐acting antivirals
GLE: glecaprevir
PIB: pibrentasvir
IDU: injecting drug use
EOT: end of treatment
SVR: sustained viral response
LDV: ledipasvir
VEL: velpatasvir
SOF: sofosbuvir
AST: aspartate aminotransferase
*γ*‐GTP: *γ*‐glutamyl transpeptidase
eGFR: estimated glomerular filtration rate
AFP: alpha fetoprotein
ADLs: activities of daily living
HCC: hepatocellular carcinoma
PWIDs: people who inject drugs

## Ethics Statement

This retrospective study was conducted in accordance with the Declaration of Helsinki as amended in 2002. Patient information was anonymized; therefore, patient identities were not discernible. We handled the data within our department′s dedicated information processing room at the Osaka General Medical Center. Information regarding this study has been published on the homepage of our hospital website. This clinical study was approved by the Institutional Review Board of the Osaka General Medical Center (No. 2021‐057, accepted on October 27, 2021).

## Conflicts of Interest

The authors declare no conflicts of interest.

## Author Contributions

Seiichi Tawara: formal analysis (lead), investigation (lead), methodology (lead), project administration (lead), supervision (lead), writing the original draft (lead), writing the review, and editing (equal). Asuka Watanabe: investigation (supporting information). Junya Yamaguchi: investigation (supporting information). Mai Omi: investigation (supporting information). Tetsuro Miyazaki: investigation (supporting). Haruka Taguchi: investigation (supporting information). Ryosuke Kiyota: investigation (supporting). Takuo Yamai: investigation (supporting). Shoichiro Kawai: investigation (supporting). Takuya Inoue: investigation (supporting information). Takayuki Yakushijin: project administration (equal), supervision (equal), and writing, review, and editing (equal).

## Funding

No funding was received for this manuscript.

## Data Availability

Data supporting the findings of this study are available upon request from the corresponding author. The data are not publicly available because they contain information that could compromise the privacy of the research participants.
